# Supplementary material for: Nurse-led psychoeducational interventions in patients suffering from schizophrenia or other psychotic disorders and their families: A scoping review protocol
Source: PLoS One. 2025 Jul 1;20(7):e0327486. doi: 10.1371/journal.pone.0327486 (PMC12212495; doi:10.1371/journal.pone.0327486)
Supplement: S1 Supplemental materiaI 1 — (DOCX) [file pone.0327486.s001.docx]

# Supplemental materiaI 1: Search strategy

1. **SCOPUS**
2. ( psychoeducation OR psycho-education OR "psychoeducational
3. program" OR "psychoeducational programme" OR "psychoeducational
4. course" OR "psychoeducational
5. intervention" ) AND ( schizophrenia OR psychosis OR psychoses OR "psychotic
6. disorder" OR "schizophrenic
7. disorder" ) AND ( nurse OR nurses OR nursing OR "nursing staff" OR "nursing
8. healthcare profession" OR "registered nurse" ) AND ( LIMIT-
9. TO ( LANGUAGE , "English" ) OR LIMIT-TO ( LANGUAGE , "Italian" ) )

# MEDLINE

1. ( psychoeducation OR psycho-education OR "psychoeducational program" OR
2. "psychoeducational programme" OR "psychoeducational course" OR "psychoeducational
3. intervention" ) AND ( schizophrenia OR psychosis OR psychoses OR "psychotic disorder"
4. OR "schizophrenic disorder" ) AND ( nurse OR nurses OR nursing OR "nursing staff" OR
5. "psychiatric nurse"[All Fields] ) Filters: English, Italian

# PsycINFO

1. **150** Results for (**Any Field**: nurse *OR* **Any Field**: nurses *OR* **Any Field**: nursing *OR* **Any**
2. **Field**: "nursing staff" *OR* **Any Field**: "psychiatric nurse") *AND* (((**Any**
3. **Field**: psychoeducation) *OR* (**Any Field**: psycho-education) *OR* (**Any Field**: "psychoeducational
4. program") *OR* (**Any Field**: "psychoeducational programme") *OR* (**Any Field**: "psychoeducational
5. course") *OR* (**Any Field**: "psychoeducational intervention")) *AND* ((**Any**
6. **Field**: schizophrenia) *OR* (**Any Field**: psychosis) *OR* (**Any Field**: psychoses) *OR* (**Any**
7. **Field**: "psychotic disorder") *OR* (**Any Field**: "schizophrenic disorder")) *AND* ((**Any**
8. **Field**: nurse) *OR* (**Any Field**: nurses) *OR* (**Any Field**: nursing) *OR* (**Any Field**: "nursing
9. staff") *OR* (**Any Field**: "psychiatric nurse"))) *AND* **Year**: 0 *To* 2025

# CINAHL

1. (nurse OR nurses OR nursing OR "nursing staff" OR "psychiatric nurse") AND
2. (((psychoeducation) OR (psycho-education) OR ("psychoeducational program") OR
3. ("psychoeducational programme") OR ("psychoeducational course") OR
4. ("psychoeducational intervention")) AND ((schizophrenia) OR (psychosis) OR (psychoses)
5. OR ("psychotic disorder") OR ("schizophrenic disorder")) AND ((nurse) OR (nurses) OR
6. (nursing) OR ("nursing staff") OR ("psychiatric nurse")))
