# Supplementary figures and images for: Nurse-led psychoeducational interventions in patients suffering from schizophrenia or other psychotic disorders and their families: A scoping review protocol
Source: PLoS One. 2025 Jul 1;20(7):e0327486. doi: 10.1371/journal.pone.0327486 (PMC12212495; doi:10.1371/journal.pone.0327486)

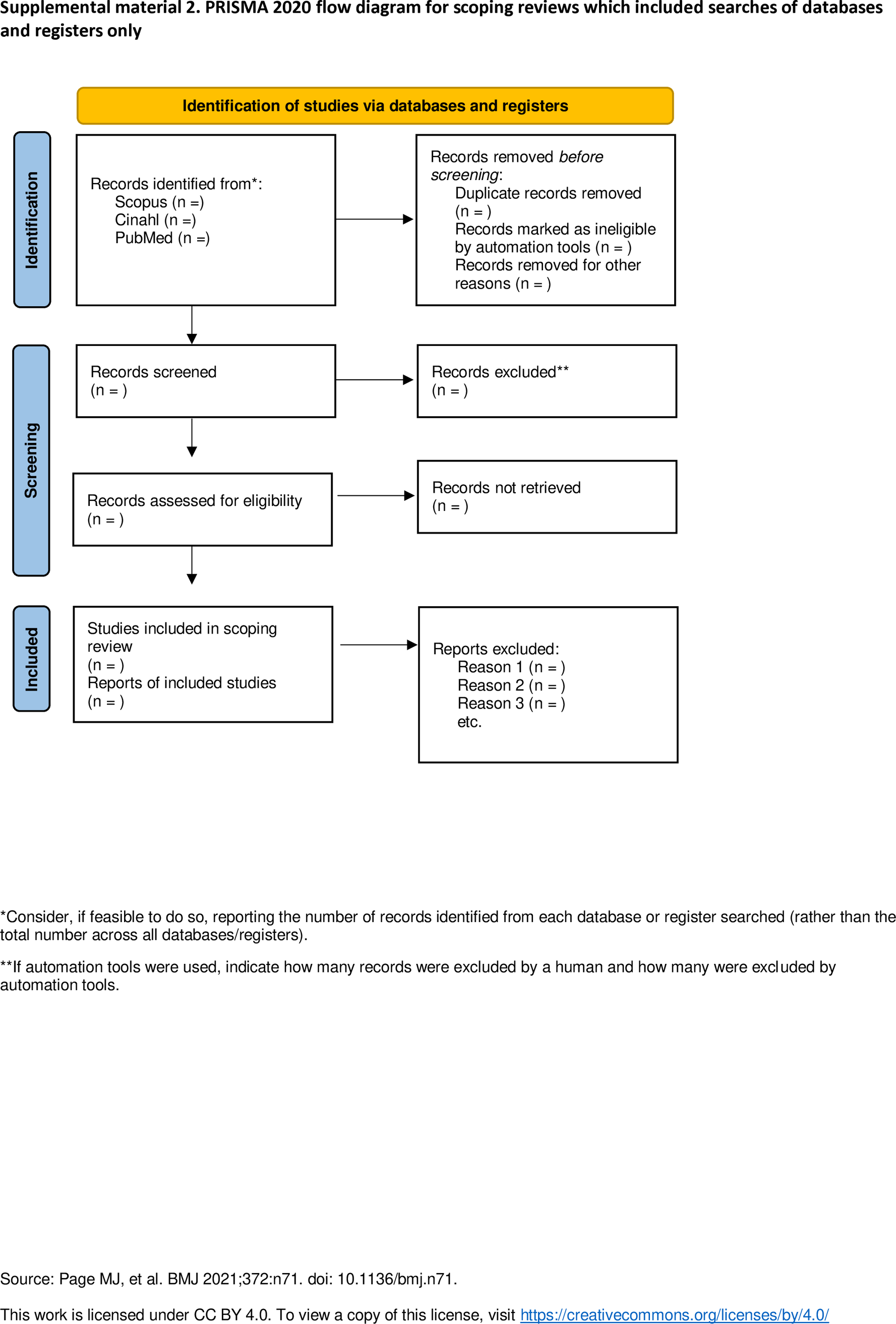

Supplement: S2 Supplemental materiaI 2 — (TIF) [file pone.0327486.s002.tif]
