# Supplementary material for: Nurse-led psychoeducational interventions in patients suffering from schizophrenia or other psychotic disorders and their families: A scoping review protocol
Source: PLoS One. 2025 Jul 1;20(7):e0327486. doi: 10.1371/journal.pone.0327486 (PMC12212495; doi:10.1371/journal.pone.0327486)
Supplement: Attachment — Submitted filename: Reviewer answers (27.05.2025).docx [file pone.0327486.s005.docx]

**Reviewer #1:** PLOS ONE REVIEW

The manuscript (ms) presents a scoping review protocol aimed at mapping existing research on nurse-led psychoeducational interventions for patients with schizophrenia or other psychotic disorders and their families. This is a relevant and important topic, as it highlights the potential role of nurses in delivering structured psychoeducational programs that may improve treatment adherence and patient outcomes. However, while the study aims to address an important gap in the literature, several sections require further clarification and refinement before I can recommend the ms for publication.

While the authors emphasize the limited clinical evidence regarding nurse-led psychoeducational interventions, it remains unclear whether there is a sufficient foundation for conducting this review. Furthermore, if the effectiveness and outcomes of these interventions are not being assessed, the value of the review needs to be better articulated.

There are inconsistencies between the research questions and the manuscript’s content:
• The data analysis section states that an improvement in well-being and quality of life is expected, yet none of the research questions focus on assessing improvements.

Sorry for the misunderstanding. We have underscored in the data analysis section that an improvement in well-being and quality of life will not be assessed in this scoping review, as this question would be more suitable for a systematic review

• The manuscript states that it will extract information on whether interventions target (a) patients and their families or (b) are specifically nurse-led, but it is unclear which research question this aligns with. Furthermore, there is no clear plan for comparing these groups or specifying relevant outcome measures.

Sorry for the misunderstanding. We have underscored in the data analysis section that an improvement in well-being and quality of life will not be assessed in this scoping review, as this question would be more suitable for a systematic review

The study design is appropriate for addressing the research question, but more details on the inclusion and exclusion criteria for studies (beyond age range and study design) should be provided, particularly regarding the selection of interventions that qualify as "nurse-led psychoeducation.

We added a section to the inclusion criteria regarding the selection process for studies on nurse-led psychoeducational interventions.

• Systematic reviews will be included but the ms does not specify whether the review itself or only primary studies within those reviews will be considered.

Sorry for the misunderstanding. We have underscored in the data analysis section that an improvement in well-being and quality of life will not be assessed in this scoping review, as this question would be more suitable for a systematic review

• The inclusion of “studies of particular interest and meeting the inclusion criteria deriving from the screening of the reference lists of the reviewed articles” is vague. The authors should define what constitutes a study of “particular interest.”

In light of the provided recommendation, we made a revision of the sentence.

While the manuscript outlines a structured approach to data extraction, several key elements remain unclear:
• There is no mention of how extracted data will be validated or cross-checked.

We added a sentence as you suggested.

• “Articles discussing data from the same study will all be read, but only the most relevant ones will eventually be included“. However, the procedure on duplicate data is not clear.

In response to your suggestion, we have rephrased the sentence to provide a more thorough elaboration on the concept.

• The extraction list will be modified and revised as necessary during the process of extracting data, but it is not clear if they will go back and re-review each article every time a modification is made.

Thank you for your suggestion. We have added a more detailed explanation to the text.

• The ms does not clarify how the impact of interventions on patient outcomes (e.g., adherence, QOL) will be assessed in the included studies. Details on how outcome variables will be analyzed or summarized should be included.

The impact of interventions on patient outcomes (e.g., adherence, QOL) will not be evaluated in the included studies. It seems that this may not be the intended purpose of a scoping review. We believe that we have addressed this in the text.

The manuscript is generally well-written, but certain sections are repetitive and could be streamlined for clarity. For example, the age range of participants is mentioned multiple times. Additionally, some terms should be revised to align with person-first language (e.g., “schizophrenic patients” should be changed to “patients with schizophrenia”).

The changes were made following your instructions.

Several linguistic and conceptual inconsistencies should also be addressed:
• .. conditions are amongst the biggest public health issues, triggering both severe human .. could be streamlined to "one of the most significant public health issues"
• "The success of the nurse-led psychosocial therapies created and delivered by of nurse-led psychoeducational therapies created and delivered by advanced psychiatric practice nurses." A simpler version could be: "The success of nurse-led psychoeducational therapies delivered by advanced psychiatric practice nurses...”
• One of the research questions is: “What are the characteristics of nurse-led intervention programs? The characteristics to be searched for are the name of the program”. It should be “Extracted” instead of “searched”.
• The headline of a section is “review question”, but it is “research question” throughout the text
• “Qualitative studies will also be considered that focus on qualitative data including, but not limited to, designs such as phenomenology, grounded theory, ethnography, qualitative description, action research and feminist research.” However, these are technically not designs, but research methods/methodologies and paradigms.
• They switch between calling it papers/studies throughout the ms

All your suggestions have been taken into consideration, and we have amended the manuscript accordingly.

Several methodological aspects require further clarification:
• The ms states that,”The strengths of this review are the use of the PRISMA methodology for selection and the synthesis of the articles and the use of three different databases (Scopus, PubMed and Cinahl), primarily carried out by people with experiences in the sector”. It is not clear what it is that are primarily carried out by people with experiences in the sector.

Thank you for the suggestion. We added a clearer sentence in the manuscript.

• It is not clear why “Standard interventions and/or consultations provided by mental health services will be excluded”?

Because the scoping review included only studies on nurse-led psychoeducative interventions that are not considered standard interventions, nor as consultations.

• While the ms states that data will be publicly disseminated, it does not specify the repository or how the data will be made accessible. Please specify the repository where the data will be stored (e.g., OSF or Dryad) and outline measures for validating extracted data to ensure reliability.

We specified the repository where the data will be stored (OSF). It is important to note that scoping reviews do not summarize findings from included sources of evidence, as this is more appropriate in the context of a systematic review.

• The ms does not include a preliminary PRISMA flow diagram, which is a key component of scoping review protocols. A diagram outlining the planned study selection process should be added

We added the PRISMA flow diagram as a supplementary file.

While the conclusions appropriately address the knowledge gap in nurse-led psychoeducational interventions, certain claims are made prematurely without supporting evidence. Specifically, the assertion that these interventions improve patient outcomes and treatment adherence is made without data to substantiate it. Additionally, the manuscript overgeneralizes the expected impact of the review on clinical practice. The decision to exclude grey literature may result in the omission of relevant studies, particularly in a field where nurse-led interventions may not be widely published in peer-reviewed journals. The potential limitations of excluding grey literature should be acknowledged and discussed.
We added a section on strengths and limitations, where we discussed the reason for the exclusion of grey literature.

The ms covers an important and timely topic, but several methodological and conceptual issues need to be addressed before it can be recommended for publication. The authors should:
1. Clarify the rationale for conducting this review and ensure alignment between the research questions and study objectives.
2. Provide more detail on inclusion/exclusion criteria, study selection, and data extraction.
3. Address inconsistencies and redundancies in terminology and writing.
4. Strengthen the methodological framework by specifying data validation methods and including a PRISMA flow diagram.
5. Moderate claims about the effectiveness of interventions and discuss the limitations of excluding grey literature.

**Reviewer #2:** This is the protocol for a scoping review that will be conducted on studies implementing nurse-led psychoeducational interventions for patients and families of patients with psychotic spectrum disorders. The protocol is of interest; below are some brief comments:

The manuscript requires revision by a native English speaker. Examples of corrections: “Primary and secondary studies pertaining to both quantitative and qualitative paradigm.” This sentence appears incomplete; “Adults suffering from s schizophrenia”; “and or” should likely be “and/or”; “they do not appear to be present to date similar studies in the literature” should be rephrased; “When appropriate, authors of papers will be contacted to request missing” is an incomplete sentence; “we aspect” should probably be “expect.”

An attempt was made to reformulate the previously mentioned sentences, with the objective of enhancing the quality of the English language.

This is a protocol for a study that has not yet been conducted, so ensure that sentences are in the correct tense (e.g., “We carried out this scoping review” should be “We will carry out”).

We rephrased the sentence.

Points 4 and 5 under Strengths and Limitations seem to imply that the study has already been conducted.

We rephrased these sections.

The in-text reference numbers appear after the period of the sentence they refer to. Ensure this is the correct format.

It is the correct form.

“The success of the nurse-led psychosocial therapies created and delivered by of nurse-led psychoeducational therapies created and delivered by advanced psychiatric practice nurses for patients who were recently identified and referred to psychiatric care (especially for patients suffering from schizophrenia or other psychotic disorders), however, has been demonstrated by a relatively small number of controlled trials and, consequently, limited clinical data.” This sentence is hard to read and contains errors.

We reformulated the sentences. Thank you for the suggestion.

In the subquestions of the review questions, I would add patient diagnoses to point 3.

We inserted it in point 3 of the review questions.

Since it is already March 2025, I would update the target dates for the various steps of the review.

We updated the target dates for the various steps of the review. Now it will be September 2025.

To ensure the validity of results, more information regarding the data extraction tool developed by reviewers is necessary, particularly concerning its validation if it is an automated tool for parts of the process not supervised by humans.

We inserted more information regarding the data extraction tool developed by reviewers, as suggested.

The time frame on which the research will focus must be specified, as per PRISMA guidelines. Up to which month in 2025 will inclusion criteria for the search apply?

Please note that the sentences concerning the adherence of our scoping review to the PRISMA guidelines have been incorporated in the text. The completion of the screening phase was scheduled for July 2025._

In the supplementary materials regarding search strings, at least one string lists a year limit up to 2023.

We corrected them.

7. PLOS authors have the option to publish the peer review history of their article ([what does this mean?](https://journals.plos.org/plosone/s/editorial-and-peer-review-process#loc-peer-review-history)). If published, this will include your full peer review and any attached files.


If you choose “no”, your identity will remain anonymous but your review may still be made public.


**Do you want your identity to be public for this peer review?** For information about this choice, including consent withdrawal, please see our [Privacy Policy](https://www.plos.org/privacy-policy).

Reviewer #1: **Yes:** lars clemmensen

Reviewer #2: No

[NOTE: If reviewer comments were submitted as an attachment file, they will be attached to this email and accessible via the submission site. Please log into your account, locate the manuscript record, and check for the action link "View Attachments". If this link does not appear, there are no attachment files.]

While revising your submission, please upload your figure files to the Preflight Analysis and Conversion Engine (PACE) digital diagnostic tool, <https://pacev2.apexcovantage.com/>. PACE helps ensure that figures meet PLOS requirements. To use PACE, you must first register as a user. Registration is free. Then, login and navigate to the UPLOAD tab, where you will find detailed instructions on how to use the tool. If you encounter any issues or have any questions when using PACE, please email PLOS at [figures@plos.org](mailto:figures@plos.org). Please note that Supporting Information files do not need this step.
